# E-learning readiness and perceived stress among the university students of Bangladesh during COVID-19: a countrywide cross-sectional study

**DOI:** 10.1080/07853890.2021.2009908

**Published:** 2021-12-10

**Authors:** Humayun Kabir, Md. Kamrul Hasan, Dipak Kumar Mitra

**Affiliations:** a Department of Public Health, North South University, Dhaka, Bangladesh; b CRP Nursing College, Savar, Dhaka, Bangladesh; c Department of Biochemistry and Molecular Biology, Tejgaon College, National University, Gazipur, Bangladesh

**Keywords:** COVID-19, e-learning, perceived stress, readiness, policymaking

## Abstract

**Background:**

The COVID-19 pandemic has compelled all educational institutions from the conventional campus-based education system to e-learning worldwide. However, adapting to this new platform, e-learning readiness may cause perceived stress among students. This study aimed to examine the association between e-learning readiness and perceived e-learning stress and the relationship between sociodemographic and e-learning related factors.

**Results:**

A cross-sectional study was employed, where 1145 e-learning enrolled university students were surveyed. The result indicated that nearly 91% of students reported moderate (76.07%) to the higher level (14.85%) of perceived e-learning stress, whereas more than half of them (58.17%) were at the sub-optimum level of readiness. Furthermore, it was found that students with the sub-optimum level of readiness compared to optimum had a significantly higher chance of reporting moderate and high level of perceived e-learning stress. Besides, parents’ highest education, residence, students’ preference in (e-learning or learning format), and having any eye problems were associated with perceived e-learning stress.

**Conclusions:**

A sudden introduction of e-learning during the COVID-19 catastrophe has brought about challenges, including the students' readiness, that might exacerbate the perceived stress level in different ways. This study reported that most of the students were at sub-optimal levels of readiness and suffered from moderate to high levels of perceived e-learning stress. The findings should integrate into the education monitoring system to enhance students' coping strategies, incite readiness, straighten, and nourish existing policies.

**Key messages**
- The moderate and higher level of e-learning stress was 76% and 15%, respectively.
- Here, 58% of students were at the sub-optimum level of e-learning readiness.
- Students’ sub-optimum level of e-learning readiness was significantly associated with the perceived moderate and high level of e-learning stress.

## Introduction

E-learning was introduced worldwide since the beginning of the twenty-first century in the paradigm of education technology, withholding the traditional teaching approach, upholding an often accessible, flexible, and personalised learning platform for the learners [1]. The sustainability of e-learning with the advancement of modern technology got prioritised in the education system at the university level [2]. E-learning is known as electronic learning, online learning, computer-based learning, or digital learning, which provides learning support using digital devices [3].

The deadly surge of the COVID-19 pandemic forced the students to stay at home in every corner of the earth and maintain social distancing. Thus, e-learning was prioritised by all sorts of academic institutions [4,5]. E-learning seemed to be an alternative way of continuing teaching-learning activities in the current crisis [6,7]. However, the arguments issued with the rapid switch of e-learning worldwide during the pandemic were compelling and whether it was free of barriers and adverse mental health outcomes like perceived stress, anxiety, depression, and students’ satisfaction [8–10]. Besides, the quality of e-learning is not unquestionable, where researchers recommended three domains: “resource management,” “educational attainment,” and “professional development and innovation,” and each of these is influential in ensuring sustainable practice [2,11]. Moreover, R. Adel reported that students’ satisfaction is vastly influenced by the quality attainment of e-learning [2].

In conventional campus-based learning, the interaction between learner and teacher usually becomes more intimate, resulting in the upheaval of motivation towards learning accompaniments [12]. Similarly, the teachers observed the learner’s mental states, even in an adverse mental health state like as stress or anxiety, provide an option to change teaching strategies, optimising learners’ success. Conversely, e-learning has various obstacles like a lack of non-verbal interaction that deter comprehensive learning practices [9,12]. M. Lahti et al., revealed that the conventional campus-based learning method is more auspicious than e-learning [13]. Notwithstanding, several studies reported that the learners might not feel advantageous with e-learning due to unexpected readiness, e.g. lower confidence, lower technological skills, and poor internet access [14,15].

In Bangladesh, e-learning started officially in all educational institutions during the surge of the COVID-19 [16]. During the pandemic, the newly introduced comprehensive e-learning methods were a reason to exacerbate mental health issues like as perceived stress [17,18]. The definition of stress varies based on study context or field, where researchers consider it as a complex phenomenon of the psychosomatic domain [19]. In general, in many life events, stress is experienced as emotional arousal that collectively threatens to alter the homeostasis of human physiology [20]. Perceived stress means how much stress an individual is going through under a specific period [21]. It does not necessarily concentrate on estimating the genres or frequencies of stressful events that happened to an individual, instead of how that person feels about the general stressfulness of their life [21].

E-learning is a unique learning approach conducted via the internet and electronic media that is relatively new experience for most Bangladeshi students. In such newness, readiness may matter on their performances. The e-learning readiness encompasses the availability of technology, use of technology, confidence, acceptance, and training need [22,23]. Furthermore, when we talk about learning, ‘learning encapsulates cognition’ upfronts autonomously [24]. It involves various processes, information, and manipulation of representations in the brain to produce a suitable response [25]. Mild to high stress might have a different impact on cognition [24]. Studies found that stress harms our brain and cognitive function, constrain the learning process [26,27]. In the context of learning, stress led to a decline in human memory performance as well [28]. In addition, adaptability risks are there as the pattern of traditional classroom education shifted to online learning through neuroplasticity helps in sudden adaptation [29]. Moreover, visual stimuli representation is enabled due to online education that is usually multi-method based requires digital multitasking and might lead to impaired call. Consequently, the load generated leads to poor processing and understanding of what is taught or said [30].

Again, the quality of comprehension, and prioritisation, and deep-level processing of incoming information might decline due to multimethod-based learning and divided attention. These might affect brain consolidation into long-term memory [30,31]. Besides, social cognitive abilities, e.g. such as empathy, teaming, and peer relationships among children, are impaired [29]. Also, online learning lacks social interactions that are necessary for their growth, development, and learning [32]. Recent studies reported students concerned with a lack of social interaction, interaction with instructors, and real-time communication among themselves [33,34].

Addressing the challenges of e-learning is crucial in the contemporary zeitgeist to implement it successfully in a developing country like Bangladesh and be prepared for different emergencies. To ameliorate the pandemic-induced crisis of the education sector, introducing e-learning is a timely approach; nevertheless, to make it more sustainable and compatible and as an eligible alternate of traditional face-to-face learning, the readiness, perceived stress, and other relevant predictors of e-learning are needed to be investigated. This study aimed to explore the association between students’ e-learning readiness and perceived e-learning stress, including the demographic and e-learning related factors potentially associated with perceived e-learning stress.

## Methods

### Study design

This cross-sectional study was conducted between December 26, 2020 and January 11, 2021.

### Study participants

The study participants were e-learning enrolled undergraduate and graduate-level university students in Bangladesh. Currently, there are 104 universities and approximately 3.2 million students enrolled [35]. However, the universities were closed during the COVID-19 pandemic, and the students were newly enrolled in e-learning. To participate in this study, the respondents met some inclusion criteria, included: (a) age more than 16 years, (b) willing to provide online consent, (c) being enrolled in e-learning mode during the pandemic for at least 30 days, and (d) the students of any courses or specialties.

### Data collection procedure

Data were collected online, followed by convenient sampling methods via “Google Form” during Bangladesh's COVID-19 campus-based learning closure period. Three patterns of question (single option, scale questions, and open questions) were selected for developing an online questionnaire. The study purpose and inclusion criteria were mentioned on the front page of the questionnaire. An online informed consent was taken before participating in this research. Out of the collected 1178 responses, completed 1145 responses were recruited for final analysis. The investigators maintained the anonymity of the participants.

### Questionnaire development

The questionnaire included the Perceived Stress Scale (PSS), e-learning readiness questionnaire, demographic characteristics, and e-learning related characteristics. The first part of the questionnaire included the demographic (age, gender, parent’s highest education, and division) and the e-learning related characteristics (prefer e-learning, family members prefer e-learning, having a private place, and having any eye problems). The second part of the questionnaire consisted of an e-learning readiness questionnaire, and the last part of the questionnaire was adopted for PSS.

### Measurement of perceived e-learning stress

The PSS is a self-reported 10 items scale used to measure students' perceived e-learning stress where total scores range from 0 to 40. However, the original PSS scale is a generalised version for measuring the degree of stress in the last month used to measure the conventional campus-based learning and e-learning related stress, which was slightly modified in this study [19,36]. Owing to reflecting the context of e-learning followed by B. Lazarevica and D. Bentz (Cronbach’s alpha = 0.83) [19], the phrasal modification was performed by adding the word “of e-learning” in the first clause of the items without changing the actual meaning of PSS. An item of PSS reads for “In the last month, how often have you found that you could not cope with all the things that you had to do?” After the modification of this item reads for “In the last month of e-learning, how often have you found that you could not cope with all the things that you had to do?” However, the items were responded to a five-point Likert scale of 0 for “Never” and 4 for “Very often”. To obtain the scores of PSS, firstly reversing the scores (e.g. 0 = 4, 1 = 3… & 4 = 0) of four positive items (items 4, 5, 7, & 8) and then summing all across the 10 items. The mild perceived stress of e-learning was considered while the score ranging from 0 to 13, moderate and high was considered while the scores ranging from 14 to 26 and 27 to 40, respectively [37]. The Cronbach’s alpha of PSS for the entire sample was 0.81, indicating good internal consistency in this study.

### Measurement of e-learning readiness

The e-learning readiness was accessed using 39 items of the perceived e-learning readiness questionnaire with a total score ranging from 39 to 195 [23]. The items were responded to a five-point Likert scale of 1 for “strongly disagree” and 5 for “strongly agree.” The questionnaire mainly focussed on five baseline components of e-learning; availability of technology (6 items), use of technology (11 items), self-confidence (12 items), acceptance (7 items), and training (3 items). The optimal level of readiness was determined at the mean score average of 3.40 of the readiness questionnaire [22,23,38]. Whereas less than the mean score average (< 3.4) indicated ‘sub-optimum’ and mean score average (≥ 3.4) or more indicated ‘optimum’ level of readiness. The internal consistency of this questionnaire was found suitable in the total sample (*α* = 0.95).

### Statistical analysis

Descriptive statistics were performed for demographic, e-learning related factors, PSS, and e-learning readiness questionnaire. The PSS and readiness questionnaire scores were presented as mean, median, standard deviation (SD), and interquartile range (IQR). A multinomial logistic regression model was fitted to find the association between e-learning readiness and perceived e-learning stress categories while controlling the demographic and e-learning related characteristics. The *p*-value < 0.05 was considered statistically significant at a 95% confidence interval. Data were analysed by using statistical software STATA-16.

### Ethics approval and consent to participate

The Ethical Review Broad of the Faculty of Life Science, North South University, approved this study (IRB No. 2021/OR-NSU/IRB/0601). The aims and objectives of the study were explained on the first page of the questionnaire. Participants provided online consent by returning the completed questionnaire

## Results

### Demographic and e-learning related characteristics of the study participants

Detailed demographic and e-learning related characteristics are presented in Table 1. The median age of the students was 22 years old, and the interquartile range was 20.8–23.11. More than three-quarters of them, 76.07% (*n* = 871), reported moderate level of perceived e-learning stress, and 9.08% (*n* = 104) reported high level of perceived stress, whereas 14.85% (*n* = 170) reported mild level of perceived e-learning stress. More than half of them, 58.17% (*n* = 666), reported sub-optimal level of readiness for e-learning, as opposed to only 41.83% (*n* = 479) of them reported optimal level of readiness. Female students were 52.40% (*n* = 600). Regarding parents’ highest education level, 59.91% (*n* = 686) of their parents were under-graduate completed. Here, 70.31% (*n* = 805) of them participated from Dhaka. More than half of them, 58.25% (*n* = 667), preferred e-learning. Less than half, 40% of their family members, preferred e-learning. The majority of them, 53.36% (*n* = 611), had private place for e-learning, and 46.03% (*n* = 527) reported having any eye problems.

**Table 1.** Descriptive statistics of demographic and e-learning related characteristics (*n* = 1145).

Characteristics	*n* (%)
Perceived stress	
Mild (0–13)	104 (9.08)
Moderate (14–26)	871 (76.07)
Higher (27–40)	170 (14.85)
E-learning readiness	
Sub-optimal (<3.4)	666 (58.17)
Optimal (≥3.4)	479 (41.83)
Median age (IQR), year	22 (20.8–23.11)
Gender	
Male	545 (47.60)
Female	600 (52.40)
Residence	
Dhaka	805 (70.31)
Other than Dhaka	340 (29.69)
Parents’ highest education	
Graduated	255 (22.27)
Under-graduate	686 (59.91)
Up to primary	204 (17.82)
Prefer e-learning	
No	478 (41.75)
Yes	667 (58.25)
Family members prefer e-learning	
No	677 (59.13)
Yes	468 (40.87)
Having a private place for e-learning	
No	534 (46.64)
Yes	611 (53.36)
Having any eye problems	
No	618 (53.97)
Yes	527 (46.03)

### Descriptive statistics of PSS and readiness questionnaire

In Table 2, the mean score of the PSS was found 21.03 (SD: 6.01). The mean score of e-learning readiness questionnaires was found 127.54 (SD: 27.05).

**Table 2.** Descriptive statistics and Cronbach alpha of PSS and e-learning readiness questionnaire (*n* = 1145).

Scale	Mean	Median	SD	IQR	Cronbach's alpha
PSS	21.03	21	6.01	18–24	0.81
E-learning readiness	127.54	127	27.05	111–145	0.95

### Association between e-learning readiness and perceived e-learning stress

In Table 3, a multinomial logistic regression was used to assess the association of e-learning readiness, demographic, and e-learning related factors with perceived e-learning stress. The reference category of the outcome variable was “mild stress,” and each of the other two (moderate and high stress) was compared to this reference group. The analysis was focussed on the association between e-learning readiness and perceived e-learning stress while controlling for age, gender, residence, parent’s highest education, preference of e-learning, family members’ preference of e-learning, having a private place, and having any eye problems. The results indicated that suboptimal level of e-learning readiness was significantly associated with both moderate (AOR = 2.29, 95% CI: 1.39–3.78) and high levels (AOR = 4.25, 95% CI: 2.32–8.09) of perceived e-learning stress compared to the students with the optimum level of readiness controlling for all other covariates. In the multivariable model, the age of the students had no significant association with the perceived e-learning stress. The female was significantly associated with high level of e-learning stress (UOR = 2.22, 95% CI: 1.35–3.66) in the bivariate model compared to the male but lost its significance in the adjusted model. Higher educational level of parents compared to primary level was more likely to perceive moderate (graduate-level study: AOR = 2.44, 95% CI: 1.27–4.72; undergraduate level study: AOR = 2.18, 95% CI: 1.26–3.76) and high level of e-learning stress (graduate-level study: AOR = 2.63, 95% CI: 1.12–6.18; undergraduate level study: AOR = 2.91, 95% CI: 1.45–5.86). Students from other than Dhaka had a 87% higher chance of reporting moderate (AOR = 1.87, 95% CI: 1.07–3.28) and a 92% higher chance of reporting high-level of e-learning stress (AOR = 1.92, 95% CI: 0.99–3.71) compared to the student from Dhaka. Students’ non-preference of e-learning was significantly associated with high-level e-learning stress (AOR = 5.69, 95% CI: 2.55–12.70). Students whose family members did not prefer e-learning were more likely to found moderate (UOR = 3.00, 95% CI: 1.94–4.63) and high (UOR = 8.93, 95% CI: 5.10–15.66) level perceived e-learning stress in unadjusted analysis. In adjusted analysis, family members’ preference was not found significant. Students who have any eye problems were more likely to report high-level e-learning stress (AOR = 2.53, 95% CI: 1.45–4.39) than students without any eye problems. We also analysed considering each subdomain as separate predictors, and the results are shown in Supplementary File 1.

**Table 3.** Multinomial logistic regression model between e-learning readiness and perceived e-learning stress among university students (*n* = 1145).

Variables	Moderate stress				High stress			
	UOR	95% CI	AOR	95% CI	UOR	95% CI	AOR	95% CI
E-learning readiness								
Sub-optimum	3.47	2.21–5.44	2.29**	1.39–3.77	11.15	6.27–19.84	4.25***	2.32–8.09
Optimum	Reference				Reference			
Age	0.93	0.83–1.04	0.98	0.87–1.11	0.81	0.71–0.93	0.90	0.77–1.05
Gender								
Female	1.26	0.83–1.89	1.02	0.65–1.59	2.22	1.35–3.66	1.29	0.73–2.25
Male	Reference				Reference			
Residence								
Other than Dhaka	2.09	1.23–3.54	1.87*	1.07–3.28	2.41	1.32–4.39	1.92	0.99–3.71
Dhaka	Reference				Reference			
Parents’ highest education								
Graduated	1.61	0.88–2.95	2.44**	1.27–4.72	1.22	0.57–2.62	2.63*	1.12–6.18
Under-graduate	1.62	0.98–2.67	2.18**	1.26–3.76	1.78	0.96–3.30	2.91**	1.45–5.86
Up to primary	Reference				Reference			
Prefer e-learning								
No	3.78	2.15–6.64	1.91	0.97–3.77	15.99	8.41–30.43	5.69***	2.55–12.70
Yes	Reference				Reference			
Family members prefer e-learning								
No	3.00	1.94–4.63	1.62	0.95–2.75	8.93	5.10–15.66	1.71	0.82–3.53
Yes	Reference				Reference			
Having a private place for e-learning								
No	1.77	1.15–2.73	1.25	0.77–2.02	3.75	2.23–6.29	1.70	0.94–3.08
Yes	Reference				Reference			
Having any eye problems								
Yes	1.65	1.07–2.53	1.43	0.91–2.23	3.41	2.04–5.70	2.53**	1.45–4.39
No	Reference				Reference			

CI, Confidence interval; UOR, Unadjusted odds ratio; AOR, Adjusted odds ratio; *P*-value: *< 0.05, **< 0.01, ***< 0.001.

## Discussion

From the beginning of 2020, COVID-19 hit Bangladesh and surged rapidly [39]. Therefore, continuing academic learning, e-learning initiated instead of all of its traditional campus-based learning activities [40]. However, e-learning related stress and stressors were documented in numerous studies worldwide during the pandemic [1,41,42]. To our knowledge, the current study is the first to discuss e-learning related to perceived stress and stressors in a developing country like Bangladesh. The study's findings may help the governments and university authorities to mitigate the academic learning gap due to e-learning stress by improving students’ level of e-learning readiness. The study's findings may also help to re-evaluate the effectiveness of e-learning exploration in Bangladesh at the post-COVID-19 catastrophe.

The current study revealed that more than three-fourths of the students perceived moderate to high level of e-learning stress. Similarly, B. Lazarevica and D. Bentz found that the students engaged in e-learning had higher perceived stress than those engaged in conventional campus-based learning [19]. Prior the pandemic's hit in Lebanon, R. Adel (2017) reported that 13% of the students perceived mild to moderate levels of e-learning stress [2]. This difference might be for the variations of geographic location, time of conduction, and availability of technologies.

A large number of the students (58.17%) were at the sub-optimal level of e-learning readiness in this study. Similarly, a high proportion of students (76.07%) having perceived moderate level of e-learning stress. There was a significant association between sub-optimal readiness and moderate to high level of perceived e-learning stress. There may be several factors related to readiness that are needed to be considered in the attainment of e-learning, for example, learner’s level of technological skills, level of participation, and successful access to the internet [38,43]. A study found that when self-efficacy in computer and internet use was at the optimum level, the students’ satisfaction towards e-learning enriched [44]. Several studies reported that the optimum outcomes of e-learning depend on the learner’s level of readiness, where the sub-optimum level of readiness may enhance distresses [45,46]. On the other hand, learning distress is inversely correlated with academic performances [47]. The sudden and prolonged shifting from campus-based learning was reported to pose e-learning stress along with the sub-optimum level of readiness, feeling of isolation, and loss of contact with peers and instructors [41,48]. L. Song et al., reported that the perceived e-learning stress depended on the quality of readiness, community engagement, and academic demands of the students [49]. However, this psychological outcome was found high in our study that might be exerted due to students’ sub-optimum level of readiness. The study findings supported by F. D. Davis, reported that the ease of use and availability of technology might upheave the learners’ perceived readiness of e-learning [50]. In addition, M. Händel et al., reported that learners enjoy e-learning platforms when they are well equipped with technology and personal skills, reducing stress, loneliness, and worries [1].

In this study, the students with the sub-optimum level of e-learning readiness perceived 2 times moderate and 4 times high level of stress compared to the optimum level of readiness. Giannopoulou et al., also observed high level of stress among students during the COVID-19 catastrophe [51]. In addition, Jowsey et al., reported that the students with a lack of technology experienced stress-related symptoms (ex: frustration, the feeling of isolation, and dissatisfaction) during e-learning [52]. Similarly, Tubaishat et al. showed that the sub-optimum level of readiness, like as poor computer or technological skills, could contribute to perceiving high level of stress among the learners [53].

In this current study, age was not found associated with perceived e-learning stress. This finding may be due to the narrow age range of the study population (IQR: 21–23 years). On the other hand, several studies reported that the senior students perceived high level of stress during remote learning [41,54–56]. In contrast, Fengfeng and Dean did not find age differences among the university students during active participation in e-learning [57]. Furthermore, gender was not found to predict perceived e-learning stress significantly. Several studies reported that females perceived higher stress level than males in e-learning and traditional learning methods [41,55,56]. According to USAID, over the last 20 years, Bangladesh achieved remarkable progress on gender equality, women’s educational enrolments, and empowerment [58]. Therefore, gender differences might not be found significant in this study.

In this current study, the parents’ highest education was found significantly associated with perceiving moderate and high levels of stress. The undergraduate and graduate levels of parental highest education were more likely to be associated with the perceived moderate and high level of e-learning stress. This finding may be because the educated parents were more alert with their child’s new learning methods, academic performances, and expected grades during the conventional campus-closing period. Similarly, J. Melby and R. Conger reported that parental highest educational attainment was positively associated with adolescents' academic performance [59]. D. Stevenson and DP. Baker reported that well-educated mothers' expectations are a bit high and have more demands on children’s academic achievements [60]. Furthermore, A. Agliata and K. Renk showed that a high parental expectation perhaps results in achievement-related stress among the learners [61]. Therefore, the higher expectation of educated parents on students’ performances might have played a role in perceiving more stress.

The students not having any private place were found significantly associated with e-learning stress in the unadjusted model. However, in the adjusted model, having a private place was not significantly associated with perceived e-learning stress. There might be a confounding between having a private place and the e-learning preference of the students in this study. According to K. Lamichhane a quiet and calm environment can reduce the learning stress levels of the students [62]. Similarly, D. Masha'al et al. reported that having a private place for e-learning reduces e-learning stress among the students [41].

Dhaka is the capital and highly infrastructured division of Bangladesh [63]. In this current study, the students who participated in e-learning from outside the Dhaka division were perceived 87% more moderate level of e-learning stress. This finding might be due to the poor e-learning infrastructures, including lack of technology availability or poor internet access. According to M. Asha et al., a poor e-learning infrastructure was reported as a stressor [64]. Therefore, to cope with e-learning stress, technology availability and use could be considered practical tools.

In this present study, non-preference students perceived 5 times more higher e-learning stress than the preferred e-learning group. H. Ilgaz and Y. Gulbahar reported numerous factors responsible for students’ preference in e-learning, including disability, physical distance, paying job, individual responsibility, and accessibility [65]. However, K. Fengfeng and K. Dean found lower preference and satisfaction with e-learning among university students in the USA [57]. A high level of stress might be found in this study for the sudden installation of a new learning method. M. Asha et al. reported that the lack of preparation for introducing a new learning method might not be preferred by many learners [64].

Furthermore, the students having any eye problems, such as the doctor advised not to stay on screen for a prolonged time, were perceived 2 times morehigh level of e-learning stress in this study. This finding might be explained by the fact that the students were generally worried about their physical limitations and academic performances on new learning methods. According to L. Pearlin, the physical limitation was considered a significant stressor in adult learners that may also influence their emotional well-being [66].

### Strengths and limitations of the study

Perhaps this was the first study that attempted to access the association between perceived e-learning stress and e-learning readiness in a low middle-income country like Bangladesh. Although assessing e-learning readiness is a sine qua non, the authors only investigated the association between e-learning readiness and perceived e-learning stress. As it was a cross-sectional study, the causal explanation was not possible. Due to the sampling technique, only the students having internet access during the COVID-19 pandemic might be included. However, the PSS is a generalised stress scale used to measure e-learning stress in this study. Alongside amid COVID-19 pandemic, besides e-learning related stress, pandemic-related stress might be there, which the authors could not assess as they were solely focussed on e-learning related stress. Hence, the authors also recommend commencing more research on e-learning in Bangladesh in a broader range to reveal numerous predictors and outcomes.

## Conclusion

The study revealed that more than two-thirds of the students perceived moderate to a high level of e-learning stress, and more than half were at sub-optimum level of e-learning readiness. Henceforth, a significant association was found between e-learning readiness and perceived e-learning stress. Therefore, the consequences might affect the students’ academic progress and performance during the pandemic-related school closure period. The study may come as handy for a balanced pedagogical system both amid and post-pandemic situations. Thus, producing evidence-based actions might mitigate the consequences of e-learning readiness like perceived stress among university students, enhance their coping strategies, upheave readiness, and strengthen educational policies.

## Recommendations

The study's findings may help the educational institutions, stakeholders, and policymakers to ensure the quality of readiness of e-learning by considering the perceived e-learning stress of learners. The authors recommend that they be more watchful towards the students’ e-learning readiness, which is the baseline of our study for establishing a sustainable online learning system that can pay off cumbersome situations like pandemics and new normal life. Further longitudinal research may be required in this broader area to recognise the psychological effects of e-learning readiness during the COVID-19 pandemic and the future ahead.

## Supplementary Material

Supplemental Material

## Data Availability

The study’s dataset can be found by the following link: https://doi.org/10.5281/zenodo.5110039

## The use of modified PSS

The modified PSS was used only for research and non-profit purposes, and it had not been used for any financial purposes.
