# Supplementary material for: E-learning readiness and perceived stress among the university students of Bangladesh during COVID-19: a countrywide cross-sectional study
Source: Ann Med. 2021 Dec 10;53(1):2305–14. doi: 10.1080/07853890.2021.2009908 (PMC8667940; doi:10.1080/07853890.2021.2009908)
Supplement: Supplemental Material [file IANN_A_2009908_SM2174.zip › Supplementary_file_1.docx]

**Table 4: Multinomial logistic regression model between e-learning readiness sub-domains and perceived e-learning stress among the university students (n= 1,145)**

| **Variables** | **Moderate stress** | | | | | | **High Stress** | | | | | | |
| --- | --- | --- | --- | --- | --- | --- | --- | --- | --- | --- | --- | --- | --- |
|  | **UOR** | **95% CI** | | **AOR** | **95% CI** | | **UOR** | **95% CI** | | **AOR** | **95% CI** | |  |
| **Availability of technology** | 0.89 | 0.86-0.95 | | 0.93** | 0.88-0.98 | | 0.77 | 0.73-0.80 | | 0.82*** | 0.77-0.88 | | |
| **Use of technology** | 0.95 | 0.93-0.98 | | 1.04* | 1.00-1.09 | | 0.90 | 0.87-0.93 | | 1.04 | 0.99-1.10 | | |
| **Self-confidence** | 0.95 | 0.92-0.97 | | 0.98 | 0.94-1.01 | | 0.91 | 0.89-0.93 | | 1.01 | 0.97-1.06 | | |
| **Acceptance** | 0.89 | 0.86-0.93 | | 0.90*** | 0.86-0.95 | | 0.81 | 0.78-0.85 | | 0.83*** | 0.78-0.89 | | |
| **Training** | 1.01 | 0.94-1.08 | | 0.87** | 0.80-0.95 | | 1.04 | 0.96-1.12 | | 0.79*** | 0.71-0.88 | | |
| **Age** |  |  | | 0.99 | 0.88-1.13 | |  |  | | 0.91 | 0.77-1.07 | | |
| **Gender** | | | | | | | | | | | | | |
| Female |  |  | | 0.95 | 0.60-1.51 | |  |  | | 1.24 | 0.68-2.24 | | |
| Male | Reference | | | | | | Reference | | | | | | |
| **Residence** | | | | | | | | | | | | | |
| Other than Dhaka |  |  | 1.77 | | | 0.99-3.16 |  | |  | 1.61 | | 0.81-3.21 | |
| Dhaka | Reference | | | | | | Reference | | | | | | |
| **Parents’ highest education** | | | | | | | | | | | | | |
| Graduated |  |  | | 3.17** | 1.59-6.33 | |  |  | | 3.30* | 1.34-8.13 | | |
| Under-graduate |  |  | | 2.53** | 1.44-4.43 | |  |  | | 3.73*** | 1.80-7.73 | | |
| Up to primary | Reference | | | | | | Reference | | | | | | |
| **Prefer e-learning** | | | | | | | | | | | | | |
| No |  |  | | 1.38 | 0.67-2.83 | |  |  | | 2.60* | 1.10-6.12 | | |
| Yes | Reference | | | | | | Reference | | | | | | |
| **Family members prefer e-learning** | | | | | | | | | | | | | |
| No |  |  | | 1.40 | 0.80-2.44 | |  |  | | 1.24 | 0.58-2.63 | | |
| Yes | Reference | | | | | | Reference | | | | | | |
| **Having a private place for e-learning** | | | | | | | | | | | | | |
| No |  |  | | 1.17 | 0.71-1.92 | |  |  | | 1.28 | 0.69-2.38 | | |
| Yes | Reference | | | | | | Reference | | | | | | |
| **Having any eye problems** | | | | | | | | | | | | | |
| Yes |  |  | | 1.37 | 0.86-2.17 | |  |  | | 2.02* | 1.14-3.60 | | |
| No | Reference | | | | | | Reference | | | | | | |

Note: CI, Confident interval; UOR, Unadjusted odds ratio; AOR, Adjusted odds ratio; P-value: *<0.05, **<0.01, ***<0.001

**Association between e-learning readiness sub-domains and perceived e-learning stress**

In **Table 4**, result shows the adjusted association between e-learning readiness sub-domains and perceived e-learning stress. The results indicated that availability of technology was significantly associated with moderate (AOR = 0.93, 95% CI: 0.88 – 0.98) and high (AOR: 0.82, 95% CI: 0.77 – 0.88) level of e-learning stress. Acceptance of e-learning was significantly associated with moderate (AOR = 0.90, 95% CI: 0.86 – 0.95) and high (AOR = 0.83, 95% CI: 0.78 – 0.89) level of stress. Similarly, training was also significantly associated with moderate (AOR = 0.87, 95% CI: 0.80 – 0.95) and high (AOR = 0.79, 95% CI: 0.71 – 0.88) level of e-learning stress.
