# Supplementary material for: E-learning readiness and perceived stress among the university students of Bangladesh during COVID-19: a countrywide cross-sectional study
Source: Ann Med. 2021 Dec 10;53(1):2305–14. doi: 10.1080/07853890.2021.2009908 (PMC8667940; doi:10.1080/07853890.2021.2009908)
Supplement: Supplemental Material [file IANN_A_2009908_SM2174.zip › Supplementary_file_2.docx]

**Most acknowledgeable Research Assistant names**

| **Name** | **Affiliation** |
| --- | --- |
| 1. Shimpi Akter | Japan Bangladesh Friendship Nursing College, Dhaka, Bangladesh |
| 1. Rawshan Ara | Prime College of Nursing College, Dhaka, Bangladesh |
| 1. Tasnova Ojifa | STS Nursing College, Dhaka, Bangladesh |
| 1. Anjan Roy Raj | Jashore University of Science & Technology, Jashore, Bangladesh |
| 1. Shyjuddin Khan | 250 Beded General Hospital, Brahmanbaria, Bangladesh |
| 1. Bipasha Akter Shopna | CRP Nursing College, Savar, Bangladesh |
| 1. Shilpe Akter | Dynamic Nursing College, Dhaka, Bangladesh |
| 1. Md. Abdul Quam | AM Nursing College, Maulvi Bazar, Bangladesh |
| 1. Md Ikbal Hossain | Begum Rabeya Khatun Chowdhury Nursing College, Sylhet, Bangladesh |
| 1. Tamanna Akter | Monno Nursing College, Manikganj, Bangladesh |
| 1. Sohel Mahmud | Tejgaon College, National University of Bangladesh, Gazipur- 1704, Bangladesh |
| 1. Afia Ayub | Tejgaon College, National University of Bangladesh, Gazipur- 1704, Bangladesh |
| 1. Easin Arafat | Tejgaon College, National University of Bangladesh, Gazipur- 1704, Bangladesh |
| 1. Nusrat Bhuiyan | Tejgaon College, National University of Bangladesh, Gazipur- 1704, Bangladesh |
| 1. Iffat Ara | Tejgaon College, National University of Bangladesh, Gazipur- 1704, Bangladesh |
| 1. Sharika Tahseen | Tejgaon College, National University of Bangladesh, Gazipur- 1704, Bangladesh |
| 1. Sunzida Akter Eva | Tejgaon College, National University of Bangladesh, Gazipur- 1704, Bangladesh |
| 1. Nowrin Sultana Tumpa | Tejgaon College, National University of Bangladesh, Gazipur- 1704, Bangladesh |
| 1. Mumtasin Islam Labib | Tejgaon College, National University of Bangladesh, Gazipur- 1704, Bangladesh |
| 1. Md. Shahjadul Kabir | Tejgaon College, National University of Bangladesh, Gazipur- 1704, Bangladesh |
| 1. Jannatun Nayeem | Tejgaon College, National University of Bangladesh, Gazipur- 1704, Bangladesh |
| 1. Amrin Islam Bini | Tejgaon College, National University of Bangladesh, Gazipur- 1704, Bangladesh |
| 1. M. M. Al-Shakib Rhidoy | Tejgaon College, National University of Bangladesh, Gazipur- 1704, Bangladesh |
| 1. Zahirul Islam Kawsar | Tejgaon College, National University of Bangladesh, Gazipur- 1704, Bangladesh |
| 1. Tasnova Ojifa | Tejgaon College, National University of Bangladesh, Gazipur- 1704, Bangladesh |
